# Oxidative stress induction by narasin augments doxorubicin’s efficacy in osteosarcoma

**DOI:** 10.1186/s40360-023-00695-6

**Published:** 2023-10-20

**Authors:** Zhaoming Han, Juguang Yang, Ping Wang, Feng Bian, Jiguang Jia

**Affiliations:** 1 Department of Orthopedics, Xiangyang No. 1 People’s Hospital, Hubei University of Medicine, 15 Jiefang Road, Fancheng District, Xiangyang, 441000, China; 2 Department of Orthopedics, Wuhan Third Hospital, Tongren Hospital of Wuhan University, Pengliuyang Road 241, Wuchang District, Wuhan, 430064 China

**Keywords:** Narasin, Doxorubicin, Synergism, Oxidative stress, Osteosarcoma

## Abstract

Complications and fata toxicity induced by chemotherapy are the main challenge for clinical management of osteosarcoma. The identification of agents that can augment the efficacy of chemotherapy at lower doses may represent an alternative therapeutic strategy. Narasin is a polyether antibiotic widely used in veterinary medicine. In this study, we show that narasin is active against osteosarcoma cells at the same concentrations that are less toxic to normal cells. This effect is achieved by growth inhibition and apoptosis induction, which is mediated by oxidative stress and damage, and mitochondrial dysfunction. The antioxidant N-acetyl-l-cysteine (NAC) abolishes the anti-osteosarcoma activity. Importantly, narasin significantly augments doxorubicin’s efficacy in both osteosarcoma cell culturing system and subcutaneous implantation mouse model. The combination of narasin and doxorubicin at non-toxic doses completely arrests osteosarcoma growth in mice. Our results suggest that the concurrent administration of doxorubicin and narasin could present a viable alternative therapeutic approach for osteosarcoma.

**Supplementary Information:**

The online version contains supplementary material available at 10.1186/s40360-023-00695-6.

## Introduction

Osteosarcoma, a prevalent bone cancer originating in mesenchymal tissue, primarily affects children and young adults [1]. This high-grade malignancy is characterized by its propensity for aggressive lung metastases, leading to a poor five-year survival rate [2]. Typical therapeutic strategies for osteosarcoma involve administering chemotherapy regimens both before and after surgical resection, aiming to prevent tumor metastasis [3]. However, substantial morbidity and mortality from chemotherapy-induced toxicity in various organs and the development of resistance challenge the improvement of clinical outcome for the osteosarcoma patients [4]. There is a need to identify agents that can augment the effectiveness of chemotherapy.

Narasin is a polyether ionophonic antibiotic isolated from *Streptomyces albus*, is widely used to treat coccidiosis and bacterial infection in poultry [5]. Narasin is a derivative of salinomycin and shares similar structure with monensin [6]. Both salinomycin and monensin are polyether ionophonic, and are found to have potent anti-cancer activities in a variety of cancers [7]. In particular, apart from differentiated bulk tumor cells, salinomycin targets cancer stem cells and tumor-initiating population to suppress tumorigenicity and reverse treatment resistance [8–10]. However, whether narasin is active against cancer remains largely unknown with only two relevant studies. One recent study demonstrates that narasin inhibits tumor metastasis and growth of ERα-positive breast cancer cells, and this is through inactivation of the TGF-β/SMAD3 and IL-6/STAT3 signaling pathways [11]. Another reveals that narasin stimulates tumor necrosis factor-related apoptosis-induced ligand (TRAIL)-mediated apoptosis in glioma cells via endoplasmic reticulum stress, CHOP-mediated DR5 upregulation and c-FLIP downregulation [12].

Using both cellular and mouse models, we systematically investigated the efficacy of narasin alone, and its combination with doxorubicin, in osteosarcoma, and attempted to reveal the underlying mechanism of narasin’s activity. We found that narasin alone is effective and selective in inhibiting osteosarcoma cells, and acts synergistically with doxorubicin in vitro and in vivo. In addition, the inhibitory effects of narasin in osteosarcoma are attributed to its ability in inducing oxidative stress and mitochondrial dysfunction.

## Materials and methods

### Cell culture and chemicals

The two human osteosarcoma cell lines were obtained from were purchased from the Cell Bank of Type Culture Collection of Chinese Academy of Sciences (Shanghai) and were cultured in RPMI 1640 medium (Hyclone) supplemented with 10% fetal bovine serum (FBS, Gibco), 100 U/ml penicillin, and 100 µg/ml streptomycin (Gibco) at 37 °C in 5% CO_2_. Human normal primary osteoblast (Lonza) was cultured using the Osteoblast Growth Medium BulletKit (Catalog No. CC-3207). Narasin was obtained from Sigma. Doxorubicin and acetylcysteine (N-acetyl-l-cysteine, NAC) were obtained from Selleck Chemicals. All were reconstituted according to manufacture’s recommendations and stored at aliquots in -20^0^ C.

### Proliferation assay

Cells were plated in 96-well plate in culturing medium and treated with narasin for 72 h. Cell proliferation was assessed using BrdU proliferation assay kit (Abcam). 20 µl of BrdU working solution was added into each well and incubated at 37 °C for 3 h. The spectrometric absorbance was measured at 490 nm.

### Flow cytometry

Cells were plated in 6-well plate in culturing medium and treated with narasin for 72 h. Cells were then detached using trypsin and suspended in staining buffer using FITC Annexin V Apoptosis Detection kit (BioLegend). Percentage of Annexin V was determined through flow cytometry on MACSQuant X (Miltenyi Biotec).

### Cellular reactive oxygen species (ROS), mitochondrial potential and ATP assays

Cells were plated in black, clear-bottom 96-well tissue culture dishes in culturing medium. After 24-hour narasin treatment, the medium was removed. For ROS measurement, ROS red dye working solution was added to each well. Fluorescent was measured at Ex/Em (520/605 nm) on Spectramax M5 microplate reader. For mitochondrial potential measurement, 100 nM TMRE and 10 µM MitoTracker Green (Life Technologies) was added. Fluorescence was measured at Ex/Em (495/525 nm) for MitoTracker green and at Ex/Em (550/580 nm) for TMRE. The ratio of the fluorescence signal of TMRE to that of MitoTracker green represents the mitochondrial potential. For ATP measurement, cell lysis buffer was firstly added, followed by adding substrate (Luciferase/Luciferin) solution. Luminescence was measured with a 1 s integration time.

### Western blotting

Treated cells were lysed with 5% sodium dodecyl sulfate (SDS) sample buffer containing protease inhibitor cocktail (Roche). Proteins were loaded to SDS polyacrylamide gel, were separated by electrophoresis, were transferred to polyvinylidene difluoride membrane, and were immunoblotted with antibodies against γ-H2AX (clone JBW301; Millipore) and α-tubulin. Primary antibodies were detected with HRP conjugated goat secondary antibody. The immunoblot was developed using the VersaDoc Gel Imaging System (BioRad).

### Cellular protein carbonylation

Protein was extracted from 10^6^ treated cells using RIPA buffer and protein carbonyl content was measured using the Protein Carbonyl ELISA kit (Enzo LifeSciences) as per manufacture’s protocol. A standard curve was prepared and absorbance was read on a Spectramax M5 Microplate reader at 450 nm.

### Lipid peroxidation

Lipid peroxidation was measured using the Lipid Peroxidation MDA Assay Kit (Abcam). Protein was extracted from 10^6^ treated cells in MDA lysis buffer and was mixed with TBA solution. After incubation, each sample was pipetted into a 96-well plate. A standard curve was prepared and absorbance was read on a Spectramax M5 Microplate reader at 532 nm.

### Mito stress assay

Cells were plated in XF24 tissue culture plate in culturing medium and treated with narasin for 24 h. Medium was changed to XF assay medium and incubated at 37 °C in a non-CO_2_ incubator. Oxygen consumption rates (OCR) were measured at 37 °C as per the standard protocol of XF24 extracellular analyzer (Seahorse Bioscience). The Seahorse XF-24 software calculated basal and maximal OCR automatically.

### Osteosarcoma growth in mice and immunohistochemistry

Animal work was approved by the Institutional Animal Care Committee of Xiangyang No.1 People’s Hospital. 8 nude mice in each group and 6 × 10^5^ cells in 0.1 mL 10x diluted Matrigel were inoculated into left flank of 4-week-old male Nu/Nu mice (Vital River Laboratories). When tumor reached ~ 100 mm^3^, mice were randomly classified into four groups: vehicle control, narasin or doxorubicin alone, the combination of narasin and doxorubicin. Narasin at 1.5 mg/kg was given via intraperitoneal injection once per day. Doxorubicin at 0.5 mg/kg was given via intraperitoneal injection twice per week. After three weeks, mice were euthanized using CO_2_ inhalation. Tumor specimens were harvested and processed for immunohistochemistry following established procedures. Sections of tumor tissue were fixed using 4% formalin (Sigma, USA). Apoptotic activity within the tumors was evaluated utilizing the ApoAlert DNA Fragmentation Assay Kit (Clonetech, USA), employing the well-recognized terminal deoxynucleotidyl transferase (TdT)-mediated dUTP nick-end labeling (TUNEL) technique. Subsequent to staining, hematoxylin was employed to visualize nuclei.

### Statistical analyses

Data are represented by means ± standard deviation. In vitro experiments were performed at least three times. Statistical analyses were performed by unpaired Student’s t test. Two-way ANOVA was used for in vivo work. P-value < 0.05 was considered as statistically significant.

## Results

### Narasin inhibits growth and survival in osteosarcoma cells and is less toxic to normal cells

**Fig. 1:**
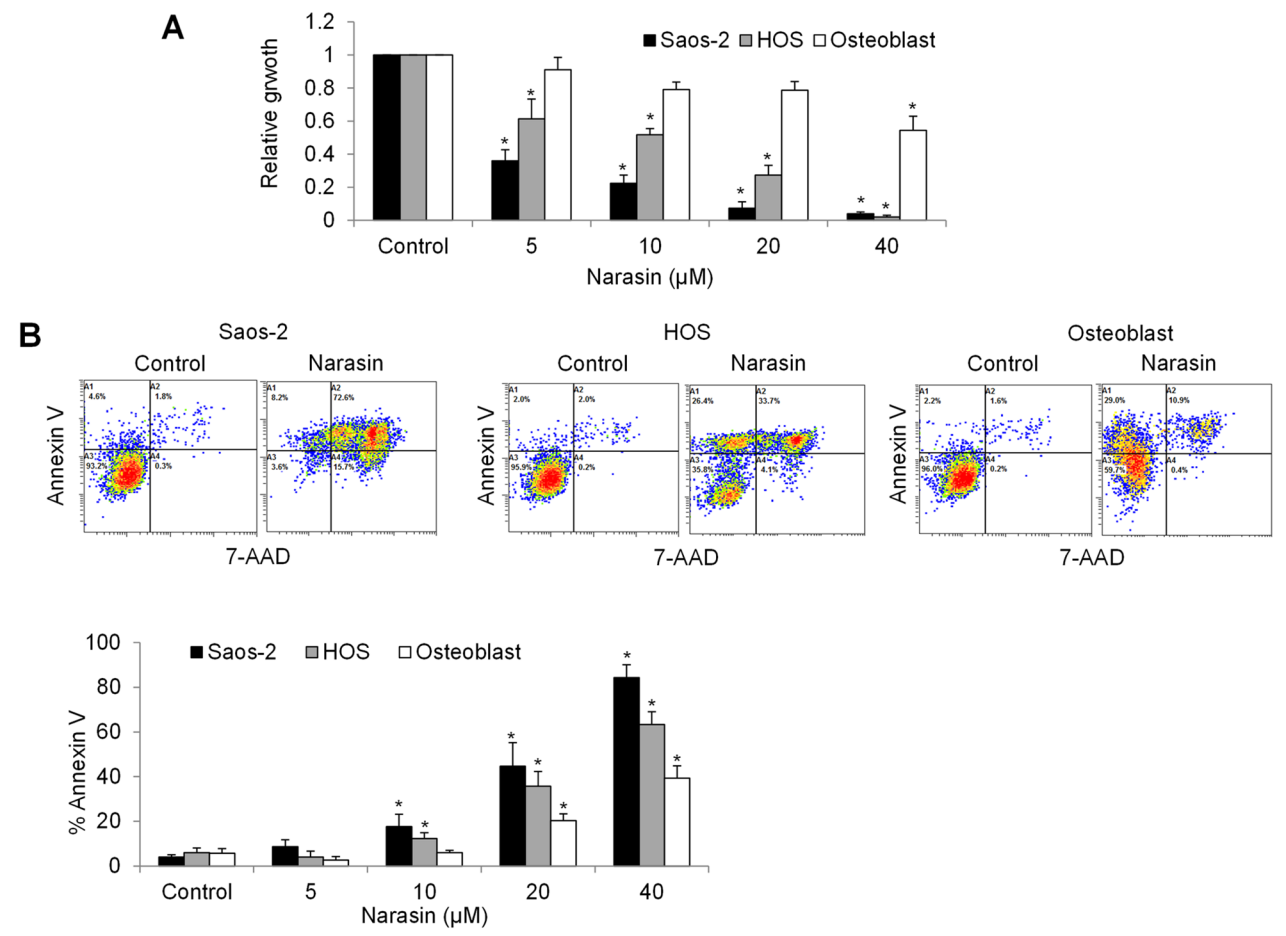
Narasin selectively inhibits growth and induces apoptosis in osteosarcoma cells. **(A)** Measurement of BrdU incorporation showed proliferation level in osteosarcoma and osteoblast cells. **(B)** Annexin V and 7-AAD double staining was used to determine cell apoptosis by flow cytometry. Annexin V(+)/7-AAD(-) and Annexin V (+)/7-AAD(+) cells were considered as apoptotic cells. Proliferation and apoptosis were measured after 72 h narasin treatment. *, p < 0.05 represents significant difference compared with cells without narasin treatment

We examined the effect of narasin on osteosarcoma cell growth and survival. We exposed two osteosarcoma cell lines for 72 h, followed by performing proliferation and apoptosis assays. Narasin at 5, 10, 20 and 40 µM decreased proliferation by 65%, 78%, 92% and 96% in Saos-2 cells, and 39%, 49%, 73% and 98% in HOS cells (Fig. 1A). In contrast, narasin at 5, 10, 20 and 40 µM decreased proliferation by 9%, 21%, 22% and 46% in normal fibroblast BJ-5ta cells. In addition, narasin at 5 µM did not affect osteosarcoma nor normal fibroblast cell apoptosis (Fig. 1B). Narasin at 10, 20 and 40 µM increased apoptosis by 17%, 44% and 63% in Saos-2 cells, 12%, 35% and 63% in HOS cells, and 6%, 20% and 39% in human normal primary osteoblast cells. These results indicate that narasin at low micromolar concentrations is effective in inhibiting growth and inducing apoptosis in osteosarcoma cells, and furthermore that narasin is less toxic to normal cells.

### Narasin significantly enhances in vitro efficacy of doxorubicin in osteosarcoma cells

**Fig. 2:**
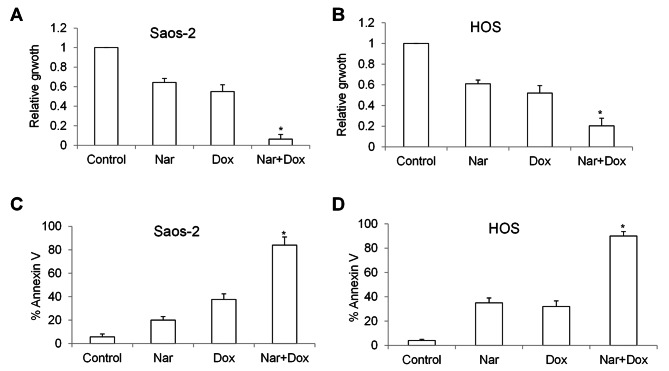
Narasin augments doxorubicin’s efficacy in osteosarcoma cells. The combination of narasin and doxorubicin results in significantly greater efficacy than single drug alone in inhibiting proliferation **(A and B)** and inducing apoptosis **(C and D)**. *, p < 0.05 represents significant difference compared with cells treated with doxorubicin alone

We next examined the combinatory effect of narasin with chemotherapy on osteosarcoma cells. Doxorubicin, one of the most active chemotherapeutic drugs used in osteosarcoma [13], was used in our combination studies. Concentration of each drug was determined based on our preliminary data that single drug alone treatment leads to < 50% growth inhibition and < 40% apoptosis induction. We found that narasin and doxorubicin alone inhibited growth by 36% and 45% in Saos-2 cells; 39% and 48% in HOS cells (Fig. 2A and B). The combination inhibited growth by 95% in Saos-2 and 80% in HOS. Narasin and doxorubicin alone induced apoptosis by 20% and 38% in Saos-2 cells; 35% and 32% in HOS cells (Fig. 2C and D). The combination inhibited growth by 84% in Saos-2 and 90% in HOS. Combination studies using other chemotherapeutic agents demonstrated that the combined treatment of narasin with either methotrexate or cisplatin led to a more pronounced inhibitory impact on the growth of Saos-2 and HOS cells (Figure S1). These results indicate that narasin significantly enhances in vitro efficacy of chemotherapy in osteosarcoma cells.

### Narasin induces oxidative damage and mitochondrial dysfunction in osteosarcoma cells

**Fig. 3:**
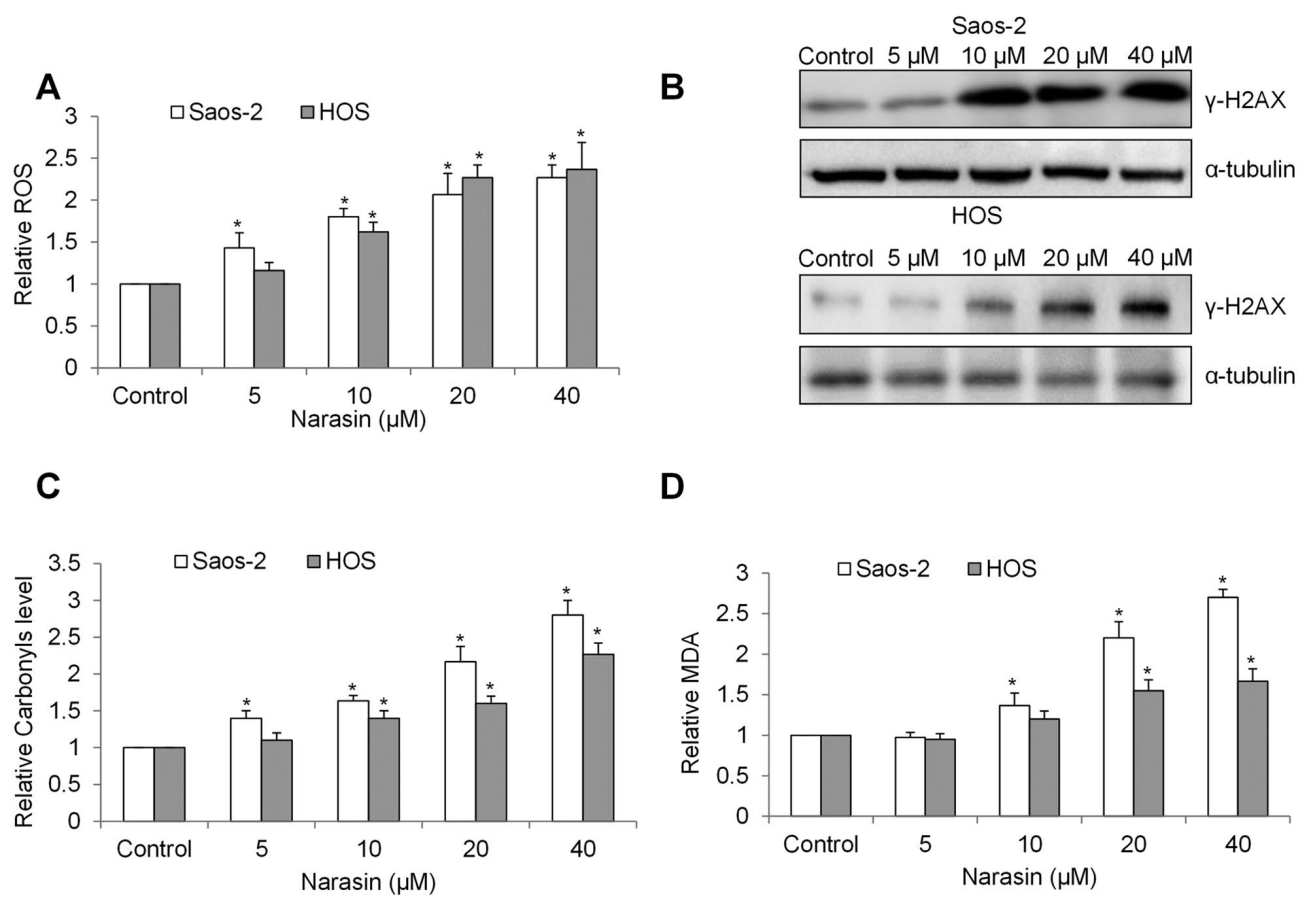
Narasin induces oxidative stress and damage in osteosarcoma cells. **(A)** Narasin significantly increase intracellular ROS level. **(B)** Western blot shows increased γ-H2AX level in narasin-treated cells. Narasin significantly increases protein carbonyls **(C)** and MDA **(D)** level. *, p < 0.05 represents significant difference compared with cells without narasin treatment

Our mechanism studies showed that narasin significantly increased ROS level in Saos-2 and HOS cells in a concentration-dependent manner (Fig. 3A). Western blot analysis of γ-H2AX, a core histone protein that is phosphorylated in response to DNA damage, was increased (Fig. 3B), suggesting oxidative DNA damage in osteosarcoma cells after narasin treatment. Consistently, protein carbonyls, a modification of proteins resulting from oxidative damage, was increased in narasin-treated cells (Fig. 3C). Malondialdehyde (MDA), an indicated of lipid peroxidation, was also increased (Fig. 3D). These results indicate that narasin induces oxidative stress and damage in osteosarcoma cells.

**Fig. 4:**
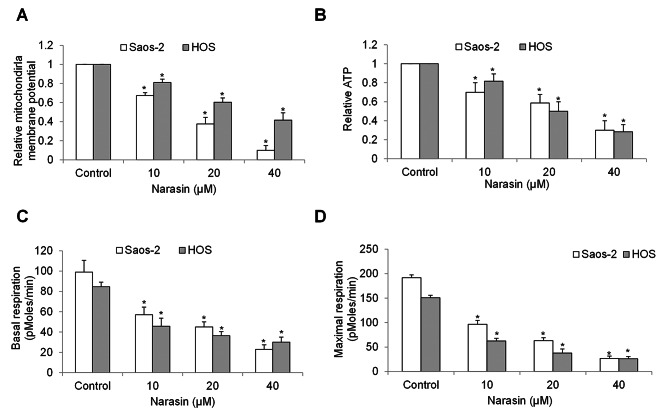
Narasin induces mitochondrial dysfunction in osteosarcoma cells. **(A)** Mitochondrial membrane potential was quantified using TMRE and MitoTracker Green. **(B)** ATP levels were measured using a luciferin/luciferase assay. The effects of narasin on the basal **(C)** and maximal **(D)** mitochondrial respiration level measured by Seahorse oxygen consumption rate (OCR) protocol. *, p < 0.05 represent significant difference compared with cells without narasin treatment

Consistent with the fact that mitochondrial dysfunction is one of the consequences of excessive ROS accumulation [14], we found that narasin significantly decreased mitochondrial membrane potential and ATP level in Saos-2 and HOS cells (Fig. 4A and B). Basal OCR, which was measured under basal condition and indicates basal mitochondrial respiration, and maximal OCR which was measured after FCCP stimulation and indicates spare respiratory capacity, were determined in cells after narasin treatment. We observed a concentration-dependent reduction in both basal and maximal mitochondrial respiration in narasin-treated cells (Fig. 4C and D).

### Narasin-induced mitochondrial dysfunction, growth arrest and apoptosis were rescued by an antioxidant in osteosarcoma cells

**Fig. 5:**
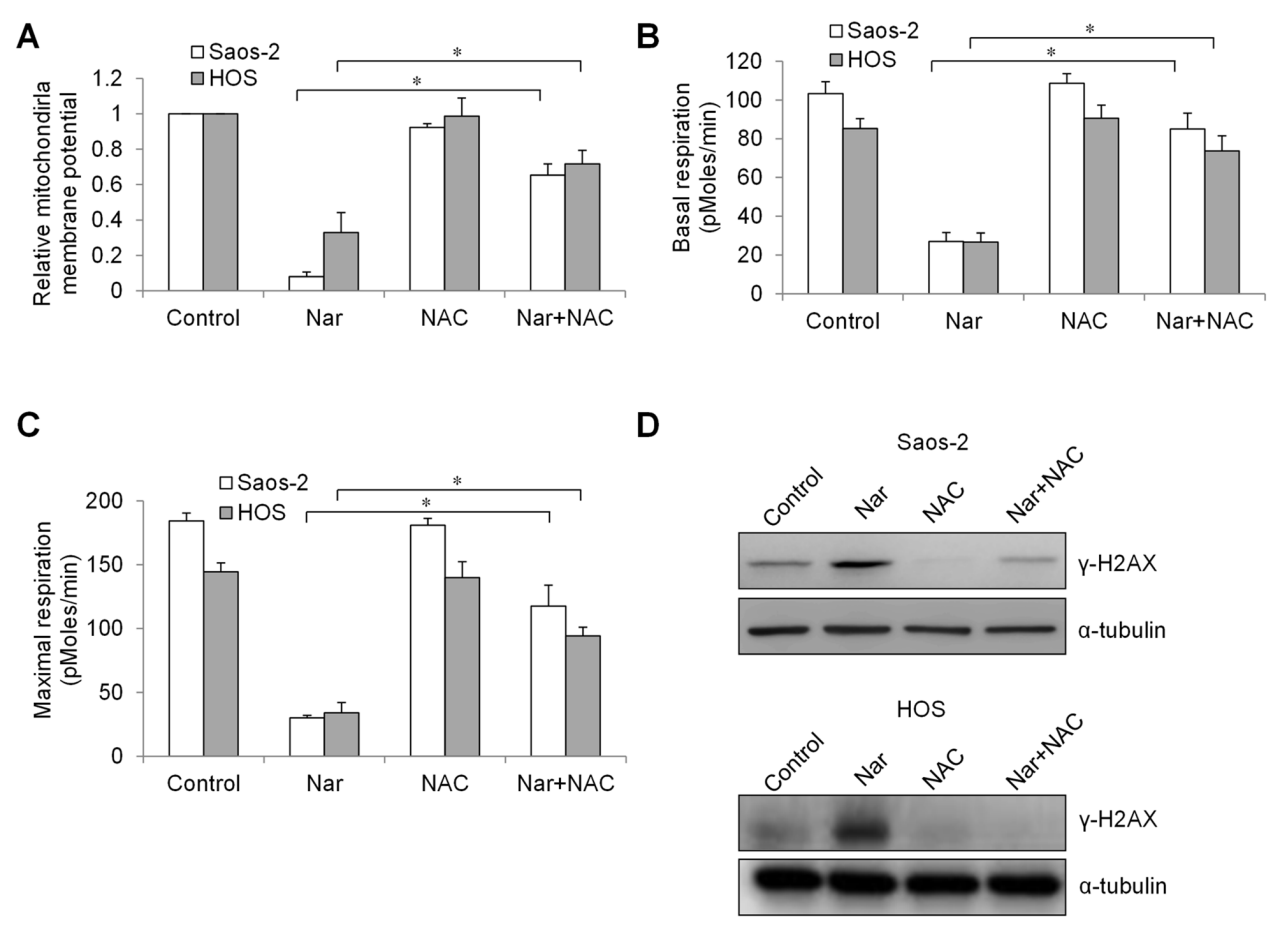
NAC rescues narasin-induced oxidative damage and mitochondrial dysfunction in osteosarcoma cells. NAC significantly reverses decreased mitochondrial membrane **(A)**, increased γ-H2AX **(B)**, decreased basal respiration **(C)** and decreased maximal respiration **(D)** induced by narasin. Osteosarcoma cells were incubated with and without NAC for 2 h, followed by treatment of narasin. *, p < 0.05 represent significant difference between narasin-treated cells with and without NAC.

**Fig. 6:**
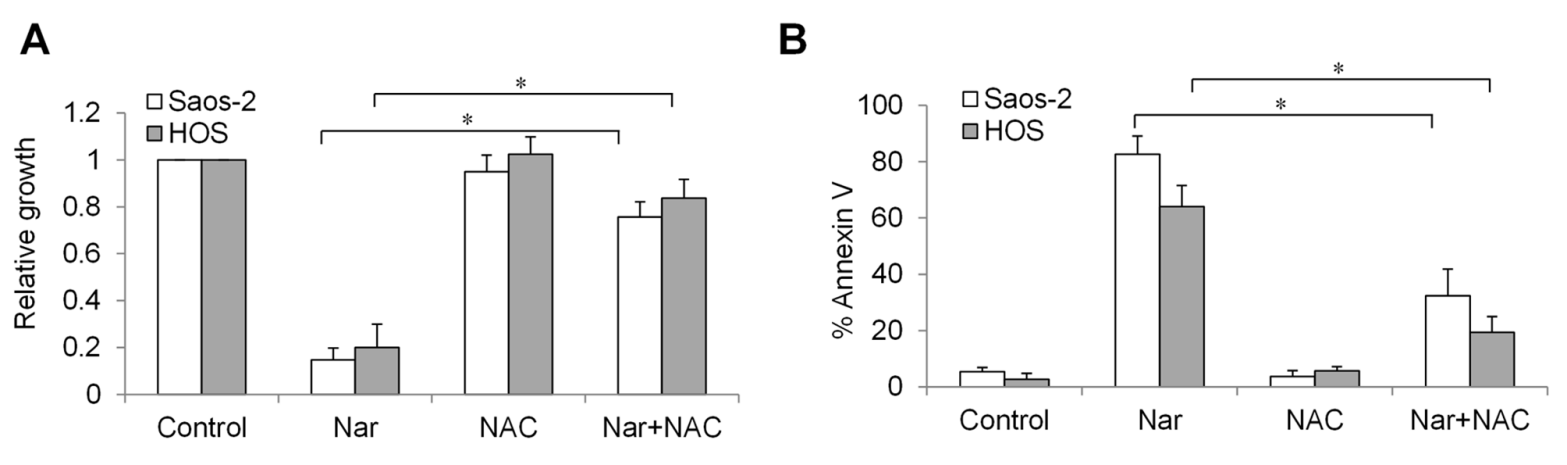
NAC rescues narasin-induced inhibition of growth and survival in osteosarcoma cells. NAC significantly reverses decreased growth **(A)** and increased apoptosis **(B)** induced by narasin. Osteosarcoma cells were incubated with and without NAC for 2 h, followed by treatment of narasin. *, p < 0.05 represent significant difference between narasin-treated cells with and without NAC.

To investigate whether oxidative stress is required for narasin’s action in osteosarcoma cells, we performed rescue studies using an antioxidant. Osteosarcoma cells were pre-treated with NAC for 2 h, followed by narasin treatment. We found that NAC pre-treatment restored mitochondrial membrane potential of narasin-treated osteosarcoma cells to almost control levels (Fig. 5A). In addition, NAC significantly reversed the inhibitory effects of narasin in decreasing basal and maximal mitochondrial respiration (Fig. 5B and C). A significant less γ-H2AX level was observed in NAC pre-treated cells in the presence of narasin compared to narasin treatment alone (Fig. 5D). Of note, NAC almost completely abolished the anti-proliferative and pro-apoptotic effects of narasin (Fig. 6).

### Narasin significantly enhances in vitro efficacy of doxorubicin in osteosarcoma cells

**Fig. 7:**
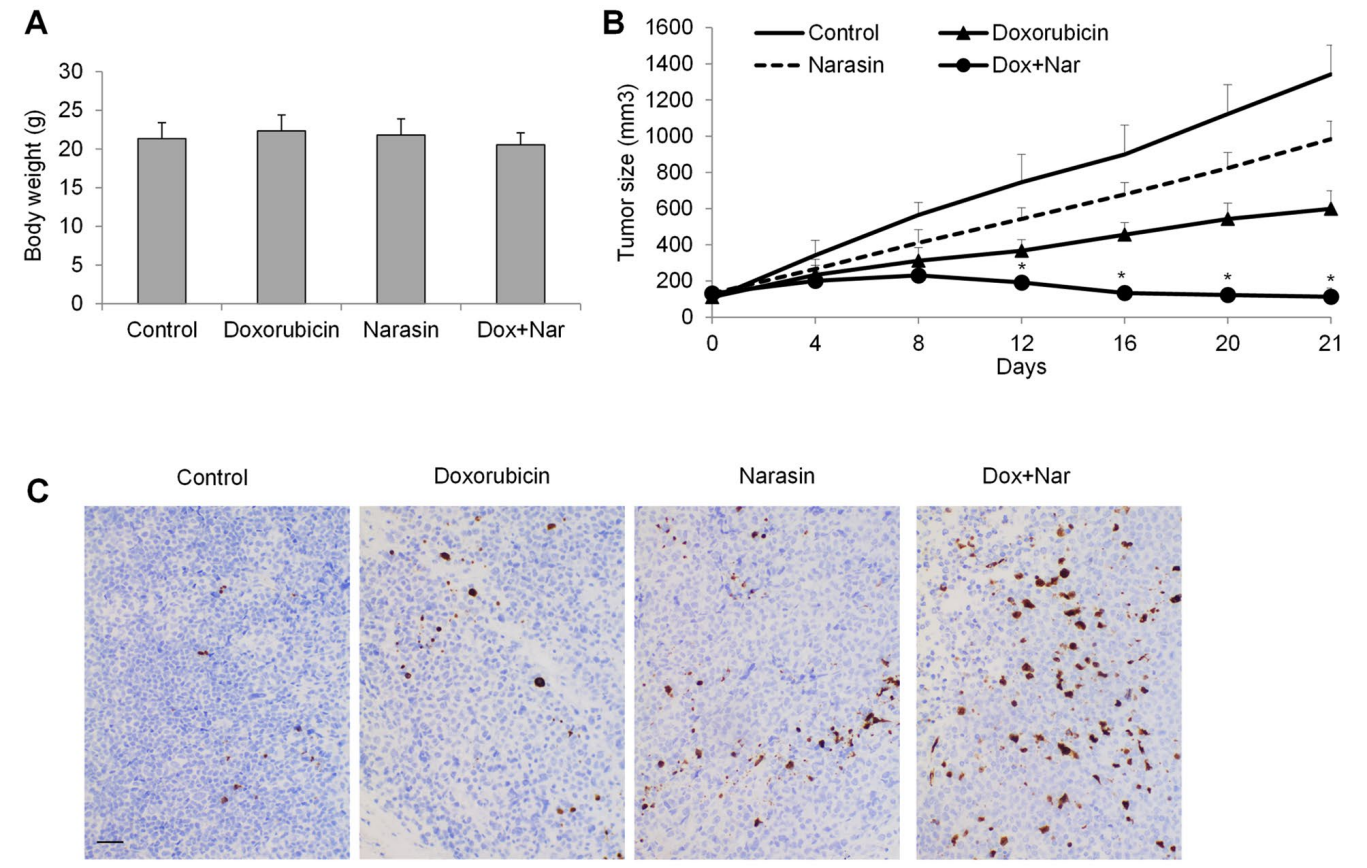
Narasin augments doxorubicin’s efficacy in osteosarcoma xenograft mouse model. Body weight **(A)** and tumor size **(B)** in mice treated with vehicle, narasin or doxorubicin alone, or combination of narasin and doxorubicin. **(C)** Representative photos of TUNEL staining depicting apoptotic tumor cells in control and drug treated groups. Scale bar represent 40 μm. *, p < 0.05 represents significant difference between doxorubicin-treated mice with and without narasin treatment

To determine whether the in vitro findings are reproducible in in vivo, we applied osteosarcoma mouse model via subcutaneous injection of Saos-2 cell/Matrigel mixture into mice. After development of palpable tumors, mice were treated with vehicle, narasin, doxorubicin alone, and the combination of narasin and doxorubicin. We assessed the general toxicity in mice subjected to drug treatment by gauging changes in body weight and scrutinizing for any deviations from the norm in physical attributes (such as fur-coat condition and edema) as well as behaviors (like vocalization, hunched posture, and shivering). We monitored tumor size (to indicate tumor growth) during the whole treatment duration. The dose of narasin employed in the combination study represented the highest concentration that did not induce toxicity in mice. This selection was based on the absence of substantial body weight reduction (Fig. 7A) or the manifestation of any anomalous physical appearances or behaviors. After 3 weeks treatment, we demonstrated that narasin slightly decreased tumor size whereas doxorubicin moderately decreased tumor size (Fig. 7B). Consistent with the in vitro finding on the synergism between narasin and doxorubicin, the combination of narasin and doxorubicin led to significant less tumor size than single drug alone. Approximately complete inhibition of tumor growth was observed throughout the whole duration of treatment in combination group. IHC staining revealed a heightened presence of apoptotic cells in tumors derived from mice subjected to combination treatment, as compared to those receiving treatment with a single drug (Fig. 7C).

## Discussion

This study provides evidence to demonstrate that (1) narasin alone is active and selective in targeting osteosarcoma cells; (2) narasin remarkably enhances in vitro and in vivo efficacy of doxorubicin; (3) treatment of narasin and doxorubicin combination is not toxic in mice; (4) narasin acts on osteosarcoma cells through inducing oxidative stress. Since osteosarcoma has complex multigenomic heterogeneity and its progression is associated with the genetic variations and instabilities [15], targeting common factor (e.g., redox homeostasis) may has advantage than specific gene or signaling pathways.

Although narasin is a polyether ionophonic antibiotic, we found that narasin at low micromolar concentrations that inhibits growth and induces apoptosis in osteosarcoma cells while is less toxic to normal fibroblast cells (Fig. 1). This finding is consistent with the previous report on the anti-proliferative and anti-survival effects of narasin in breast cancer [11]. In addition, narasin significantly augments the inhibitory effects of doxorubicin, cisplatin and methotrexate in osteosarcoma cell growth (Fig. 2 and Fig. S1). We are the first to reveal that narasin displays selective anti-cancer activity and acts synergistically with chemotherapy.

These in vitro findings are further confirmed in our in vivo studies that the combination of narasin and doxorubicin results in remarkably higher efficacy than doxorubicin alone in inhibiting osteosarcoma growth without causing significant toxicity in mice (Fig. 7). Substantial evidence shows that salinomycin selectively targets the cancer stem cells and tumor-initiating cells [9, 16, 17]. As being a derivate of salinomycin, narasin is likely to exert anti-cancer activities through targeting cancer stem cells or tumor-initiating cells. Tumor formation assay and anchorage-independent colony formation assay should be applied in the future to examine the effects of narasin on osteosarcoma stem cells and initiating cells. The validation of combinatory effects of narasin with cisplatin and methotrexate using in vivo osteosarcoma models should be conducted to confirm the synergy between narasin and chemotherapeutic agents. The combination therapy elucidated in our study might not be universally applicable across all grades and variants of osteosarcoma. Rather, our preclinical discoveries establish a proof-of-concept, showcasing the potential of combining narasin with chemotherapy for osteosarcoma treatment. To substantiate the combined efficacy and safety, clinical trials are imperative. We posit that initial recruitment for these clinical investigations should prioritize patients with advanced osteosarcoma stages, particularly those demonstrating elevated levels of mitochondrial biogenesis.

Narasin inhibits TGF‑β/SMAD3 and IL‑6/STAT3 activation in breast cancer cells [11]. Our mechanism studies show that narasin induces oxidative DNA, protein and lipid damage in osteosarcoma cells. As a consequence of oxidative and mitochondrial dysfunction (Figs. 3 and 4). Antioxidant reverses the inhibitory effects of narasin (Figs. 5 and 6). All these indicate that oxidative stress induction contributes to narasin’s effects in osteosarcoma cells. Oxidative stress has garnered attention in osteosarcoma as therapies targeting oxidative stress selectively eliminate cancer cells [18, 19]. Although high level of ROS has been detected in many cancers which promotes cancer growth and survival, many agents that lead to excessive ROS induce cancer cell death [20, 21]. Monensin which demonstrates similar structure as narasin has been reported to inhibit anaplastic thyroid cancer and prostate cancer via inducing oxidative stress [22–24]. Our work and others suggest that oxidative stress might be the target of polyether ionophonic antibiotic in cancer cells.

Similar to osteosarcoma cells, we observed a significant rise in intracellular ROS and γ-H2AX levels induced by narasin treatment in osteoblast cells as well (Figure S2A and B). In addition, narasin treatment in osteoblast cells resulted in a substantial reduction in basal and maximal OCR levels (Figure S2C and D). Interestingly, we noted that basal ROS and OCR levels are significantly lower in osteoblast cells compared to Saos-2 and HOS (Figure S3A and B). This is consistent with Chen et al’s work that osteoblast cells display decreased mitochondrial biogenesis and baseline oxygen consumption compared to osteosarcoma cells [25]. We also noted that osteoblast cells exhibited a lower ROS level in comparison to osteosarcoma cells (Figure S3C). Eleni et al. reported that ROS-low leukemia cells are dependent on oxidative respiration rather than glycolysis for energy generation than ROS-high leukemia cells [26]. Hence, we posit that the heightened susceptibility of osteosarcoma cells to narasin, relative to osteoblast cells, could be attributed to osteosarcoma cells exhibiting a greater dependence on mitochondrial functionality, in contrast to osteoblast cells. This speculation is supported by previous research on the heightened metabolic activity and reliance on mitochondrial function in tumor cells in comparison to their non-malignant counterparts [27]. AMPK activity responds to ATP levels and hence provides a direct and gauge of cellular energy status [28]. AMPK activation will inhibit the mammalian/mechanistic target of rapamycin (mTOR) signaling which regulates mitochondrial mass and functions [29]. We speculate that mTOR and AMPK pathways might be involved in the downstream of narasin’s action.

In conclusion, our study demonstrates the potential of narasin to augment doxorubicin’s efficacy in preclinical osteosarcoma models and highlights the therapeutic value of targeting oxidative stress in osteosarcoma.

### Electronic supplementary material

Below is the link to the electronic supplementary material.

Supplementary Material 1

## Data Availability

The raw data supporting the conclusions of this article are available from the corresponding author upon request.
