# Supplementary material for: Oxidative stress induction by narasin augments doxorubicin’s efficacy in osteosarcoma
Source: BMC Pharmacol Toxicol. 2023 Oct 20;24:56. doi: 10.1186/s40360-023-00695-6 (PMC10588065; doi:10.1186/s40360-023-00695-6)
Supplement: Supplementary file 1 — Supplementary Material 1 [file 40360_2023_695_MOESM1_ESM.docx]

**Oxidative stress induction by narasin augments doxorubicin’s efficacy in osteosarcoma**

Zhaoming Han, Juguang Yang, Ping Wang, Feng Bian, Jiguang Jia

**Figure S1: Narasin augments efficacy of chemotherapeutic agents in osteosarcoma cells.** The combination of narasin with methotrexate (MTX) or cisplatin (Cis) results in significantly greater efficacy than single drug alone in inhibiting proliferation of Saos-2 (A) and HOS (B) cells. *, p<0.05 represents significant difference compared with cells treated with single drug alone.

**Figure S2: Narasin induces oxidative stress and damage, and mitochondrial dysfunction in osteoblast cells.** (A) Narasin significantly increase intracellular ROS level. (B) Western blot shows increased γ-H2AX level in narasin-treated osteoblast cells. The effects of narasin on the basal (C) and maximal (D) mitochondrial respiration level measured by Seahorse oxygen consumption rate protocol. *, p<0.05 represents significant difference compared with cells without narasin treatment.

**Figure S3: Mitochondrial respiration and intracellular ROS levels of osteosarcoma and osteoblasts cells.** Relative baseline mitochondrial basal OCR (A), maximal OCR (B) and intracellular ROS (C) in Saos-2, HOS and Osteoblast (N) cells. *p<0.05, represents significant difference between osteosarcoma and osteoblast cells.


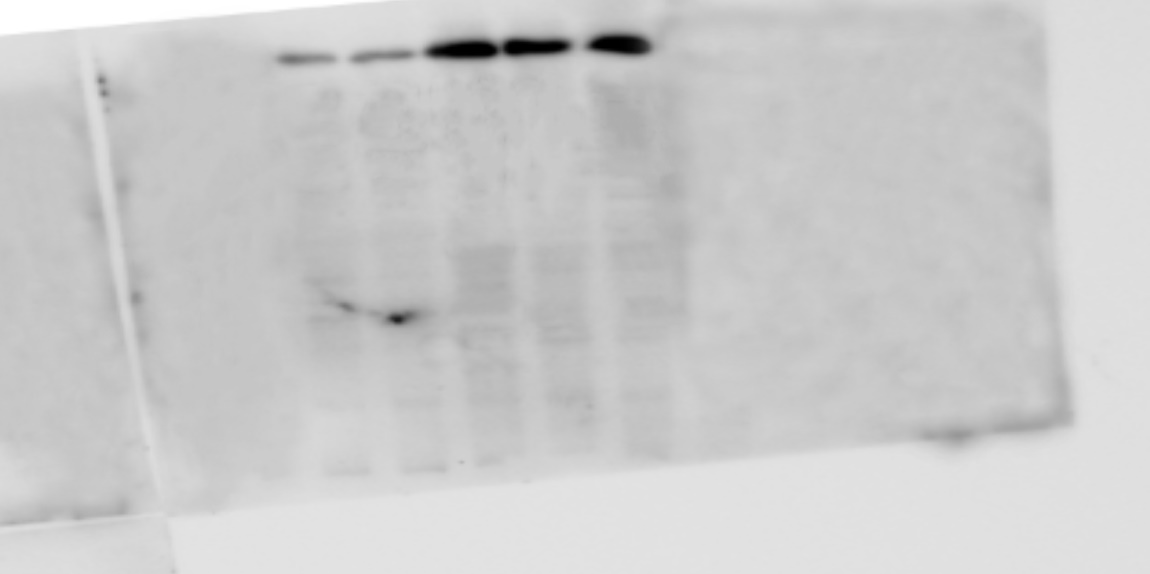


Uncropped gel for γ-H2AX shown in Figure 3A Saos-2

Uncropped gel for α-tubulin shown in Figure 3A Saos-2

Uncropped gel for γ-H2AX shown in Figure 3A HOS

Uncropped gel for α-tubulin shown in Figure 3A HOS

Uncropped gel for γ-H2AX shown in Figure 5D Saos

Uncropped gel for α-tubulin shown in Figure 5D Saos

Uncropped gel for γ-H2AX shown in Figure 5D HOS

Uncropped gel for α-tubulin shown in Figure 5D HOS

Uncropped gel for α-tubulin shown in Figure S2B γ-H2AX

Uncropped gel for α-tubulin shown in Figure S2B α-tubulin
